# Left behind and left out: The impact of the school environment on young people with continence problems

**DOI:** 10.1111/bjhp.12284

**Published:** 2017-12-11

**Authors:** Katie Whale, Helen Cramer, Carol Joinson

**Affiliations:** ^1^ Centre for Child and Adolescent Health School of Social and Community Medicine University of Bristol UK; ^2^ Centre for Academic Primary Care School of Social and Community Medicine University of Bristol UK

**Keywords:** children, education, incontinence, paediatric, paediatric incontinence, qualitative, school, self‐management, young people

## Abstract

**Objectives:**

To explore the impact of the secondary school environment on young people with continence problems.

**Design:**

In‐depth qualitative semi‐structured interviews.

**Methods:**

We interviewed 20 young people aged 11–19 years (11 female and nine male) with continence problems (daytime wetting, bedwetting, and/or soiling). Interviews were conducted by Skype (*n *= 11) and telephone (*n *= 9). Transcripts were analysed using inductive thematic analysis.

**Results:**

We generated five main themes: (1) Boundaries of disclosure: friends and teachers; (2) Social consequences of avoidance and deceit; (3) Strict and oblivious gatekeepers; (4) Intimate actions in public spaces; and (5) Interrupted learning.

**Conclusion:**

Disclosure of continence problems at school to both friends and teachers was rare, due to the perceived stigma and fears of bullying and social isolation. The lack of disclosure to teachers and other school staff, such as pastoral care staff, creates challenges in how best to support these young people. Young people with continence problems require unrestricted access to private and adequate toilet facilities during the school day. There is a need for inclusive toilet access policies and improved toilet standards in schools. Addressing the challenges faced by young people with continence problems at school could help to remove the barriers to successful self‐management of their symptoms. It is particularly concerning that young people with continence problems are at higher risk of academic underachievement. Increased support at school is needed to enable young people with continence problems to achieve their academic potential.

**Statement of Contribution**

***What is already known on this subject?***

- Continence problems are among the most common paediatric health problems
- Self‐management of continence problems requires a structured schedule of fluid intake and bladder emptying
- Inadequate toilet facilities and restricted access make it difficult for young people to manage their incontinence

***What does this study add?***

- Improvement is needed in teacher understanding of the needs of young people with continence problems
- Young people are reluctant to disclose continence problems due to perceived stigma and fear of social isolation
- Young people with continence problems may be at increased risk of academic underachievement

## Background

Continence problems are among the most common paediatric health problems, and it is commonly believed that they resolve with age. Epidemiological studies, however, have found that continence problems often persist into adolescence with urinary incontinence occurring in 2–3% and soiling occurring in 1–1.5% (Bakkar, van der Sprundel, van der Auwera, van Gool, & Wyndaele, 2002; Hellström, Hansson, Hansson, Hjaslmas, & Jodal, 1995; Heron, Grzeda, von Gontard, Wright, & Joinson, 2017; Swithinbank, Brookes, Shepherd, & Abrams, 1998; van der Wal, Benninga, & Hirasing, 2005). There is evidence from questionnaire‐based studies that continence problems in young people are associated with high levels of psychosocial problems (Feehan, McGee, Stanton, & Silva, 1990; Fergusson & Horwood, 1994; Grzeda, Heron, von Gontard, & Joinson, 2017), but there is a lack of in‐depth qualitative research. Evidence‐based knowledge of the experiences of young people with continence problems is needed to improve support for this vulnerable group.

The school environment is likely to present particular challenges for young people with continence problems because management of their symptoms during the school day often requires adherence to a structured regime of frequent toilet trips, regular fluid intake, and medication (Maternik, Krzeminska, & Zurowska, 2015; Burger *et al*., 2013; von Gontard, 2012a). Effective management of continence problems by young people at secondary school could be hampered by the increased complexity in timetabling, having multiple teachers, greater academic pressures, and adjustment to a new set of behavioural and social rules (Hanewald, 2013; Waters, Lester, Wenden, & Cross, 2012). Failure to successfully adjust to the secondary school environment can not only be detrimental to academic performance, but can also have adverse impacts on well‐being and mental health (Zeedyk *et al*., 2003).

Work conducted outside the United Kingdom has highlighted the challenges faced by school children in relation to toileting. Previous qualitative research with a general population of Swedish schoolchildren aged 6–16 years found that they often avoid school toilets due to lack of adequate facilities, offensive smells, and lack of security, and some children reported that they would rather endure physical discomfort of withholding rather than the psychological and social discomfort of using the toilet (Lundblad & Hellstrom, 2005). Qualitative work with Swedish schoolchildren aged 8–14 currently receiving treatments for functional bladder problems reported experiencing conflicting rules about toilet access, fears that peers and/or teachers will discover they have a continence problem, and difficulty reconciling toileting recommendations with their psychological needs (Lundblad, Berg, & Hellstrom, 2007). A questionnaire‐based study of US school children aged 6–19 years with chronic illness resulting in incontinence found that they had greater than expected rates of absenteeism from school (Filce & LaVergne, 2015). Two UK‐based reviews of experiences of school toilets in general population samples of schoolchildren found that they frequently express dissatisfaction with toilet facilities, they lack privacy, and they are commonly reported as locations for bullying and intimidation (Burton, 2013; Vernon, Lundblad, & Hellstrom, 2003).

To date, there has been no qualitative work on the school experiences of young people with continence problems in the United Kingdom. Previous literature on other chronic health problems in children has reported adverse impacts on schooling, both in terms of participation and educational attainment, higher rates of absenteeism, and higher levels of distractibility (Brandstaetter, Leifgren, & Silkworth, 2005; Gortmaker, Walker, Weitzman, & Sobel, 1990; Lowe, 2005). Adolescents with chronic health problems often miss part of or whole lessons resulting in reduced teacher contact and learning time and subsequent decreases in understanding and progress (Filce & LaVergne, 2015; Caldwell *et al*., 1997; Carroll, 2010; Needham, Crosnoe, & Muller, 2004). The aim of this study was to explore the experiences of young people with continence problems at secondary schools in the United Kingdom. As this topic has yet to be explored within the UK population, a qualitative approach focused on experience was deemed the most appropriate. Qualitative work offers the flexibility to explore this topic and better understand the different ways in which the school context impacts young people with continence problems.

## Method

Participants were recruited through five secondary care paediatric continence clinics (four in England and one in Scotland), and through an advertisement on a paediatric continence charity website – The Children's Bowel and Bladder Charity (ERIC, http://www.eric.org.uk).

Eligible participants were between 11 and 20 years, currently experiencing continence problems (bedwetting, daytime wetting, or soiling) or who previously experienced these problems after the age of 10, and able to speak and understand English.

Participants attending paediatric continence clinics were given a study information pack by their clinician. Those recruited through the advertisement were sent an information pack by the research team. The researcher contacted all interested participants by phone to describe the study and answer any questions.

Ethical approval was given by the National Research Ethics Service Committee South West – Central Bristol (14/SW/0059).

In‐depth semi‐structured interviews were conducted with 20 young people between February 2015 and January 2016 (see Table 1 for participant characteristics). Participants were given the option to be interviewed by telephone, Skype, or face to face (for participants within a 40‐mile radius of Bristol). Among the participants, 11 were interviewed by Skype and nine by telephone. No participants were interviewed face to face (those given this option preferred to be interviewed by Skype or telephone). A flexible topic guide was devised to assist questioning during interviews. This was developed through consultations with the paediatric continence charity ERIC, clinicians, patient representatives, and young people within the age range sampled. The topic guide helped to ensure that primary issues were covered across all interviews, but it did not dictate data collection. It covered topics such as attending appointments, treatment experiences, school or work, and thoughts and feelings about their continence problem (see Appendix 1 for the full topic guide). Due to the exploratory nature of the study, the interviews were semi‐structured. The topic guide was used as a starting point for discussion, with the flexibility to discuss novel areas introduced by the participants.

**Table 1:** Participant characteristics

Participant ID no.	Gender	Age	Continence problem	Method of interview	Organic or non‐organic continence^a^
1	M	12	Night wetting	Skype	Non‐organic
2	M	14	Day and night wetting	Telephone	Non‐organic
3	F	18	Daytime wetting	Skype	Non‐organic
4	F	14	Daytime wetting	Skype	Organic (kidney defect)
5	F	11	Day and night wetting	Skype	Non‐organic
6	M	15	Day and night wetting	Skype	Non‐organic
7	M	11	Night wetting	Telephone	Non‐organic
8	M	11	Night wetting	Telephone	Non‐organic
9	M	11	Day and night wetting	Telephone	Non‐organic
10	F	16	Night wetting	Skype	Non‐organic
11	M	14	Daytime wetting	Skype	Non‐organic
12	M	19	Night wetting	Telephone	Non‐organic
13	F	11	Soiling	Skype	Non‐organic
14	F	12	Soiling	Skype	Organic (anal stenosis)
15	F	14	Daytime wetting	Skype	Organic (cerebral palsy)
16	F	15	Daytime wetting	Telephone	Non‐organic
17	M	11	Soiling	Telephone	Non‐organic
18	F	17	Night wetting	Telephone	Non‐organic
19	M	13	Soiling	Telephone	Non‐organic
20	M	15	Day and night wetting	Skype	Non‐organic

Note

a Organic incontinence is that which has a neurological, structural, or anatomic cause. Non‐organic incontinence has no underlying organic cause.

**Figure 1:**
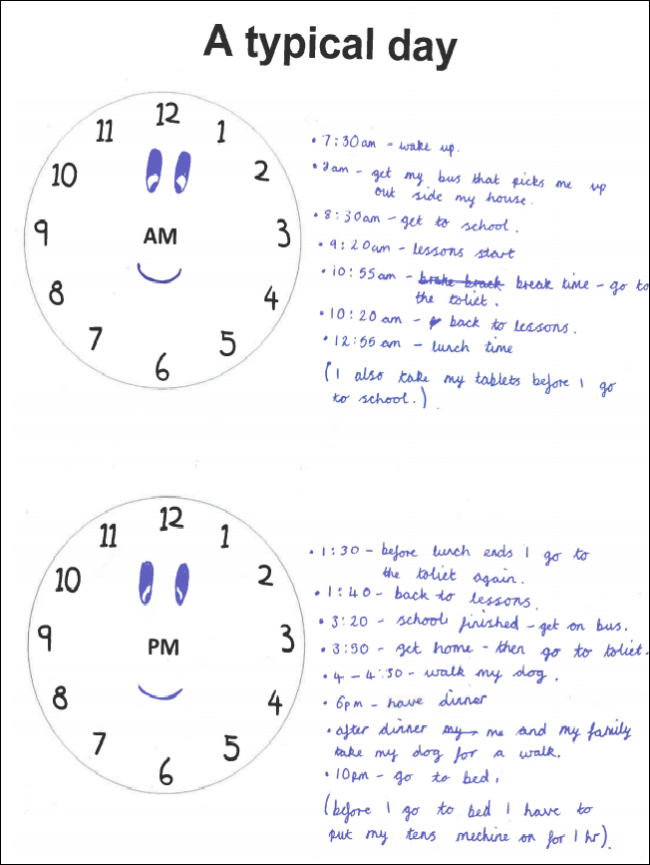
Example page of activity pack. [Colour figure can be viewed at http://wileyonlinelibrary.com]

An arts‐based participatory approach was used in the interviews. This approach is considered appropriate for children and young people as it provides additional narratives through which personal experiences can be explored (Carter & Ford, 2013). A participant activity pack was developed for use prior to and during the interviews containing a graphic representation of each possible topic area, allowing the participants to write or draw their thoughts if they wished to. Participants were sent the pack in advance of their interview and were given a verbal explanation of how it could be used during the initial phone call. Figure 1 shows an example of how the pack was used.

All participants provided written informed consent, for ages 16 and above, or parental written consent and child assent for those below 16 years. Interviews were conducted by one researcher (KW) and lasted between 34 and 99 minutes (mean 65 minutes). Data collection and analysis were conducted in parallel after completion of the first five interviews. Early analysis was used to refine the topic guide and to further explore emerging areas of interest. For example, early interviewees talked about using health or access cards at school, something which the authors had not been aware of. This was then added as a prompt in the school section of the topic guide. Interviews were audio recorded, fully transcribed, and imported into the software package NVivo10. Inductive thematic analysis was carried out following guidelines of Braun and Clarke (2006). Following completion of the first five interviews, each transcript was read and the data were free coded by hand across all transcripts. A selection of three transcripts was also independently free coded by the study team (CJ and HC). Codes were discussed and compared with all members of the team in order to further refine coding and to maximize rigour (Tracey, 2010). An initial set of agreed codes were set up within the NVivo10 database, and any new codes identified from further interviews were discussed within the team and added to the coding tree. The authors and wider researcher team brought very different perspectives to the analysis. KW had a qualitative and psychological background, CJ had a quantitative and epidemiological background, HC had a qualitative and anthropological background, and other members of the team had extensive expertise as paediatricians and specialist incontinence clinicians. A significant amount of the interview data focused on young people's experiences of school with several strong themes generated.

### Sample description

In total, 20 interviews were carried out with children and young people aged 11–19, 17 of whom were recruited through paediatric continence clinics and three through the ERIC web advertisement. Table 1 provides a full overview of participant characteristics. Seventeen participants were in full‐time education, two attended sixth form college, and one had recently begun university.

## Results

Five themes relating to school experiences were generated from the data:

1. Boundaries of disclosure: (a) friends and (b) teachers
2. Social consequences of avoidance
3. Strict and oblivious gatekeepers
4. Intimate actions in public spaces
5. Interrupted learning

### 1a. Boundaries of disclosure: friends

Deciding whether or not to disclose their continence problem to friends and peers was a very important decision for participants. Many felt strongly that they did not want their friends to know due to fears of being stigmatized, bullied or teased, or, because of this, felt too embarrassed to talk about it.

No. I couldn't. I don't know, I just couldn't. I know they're my best friends and I've been friends with them since I started high school and they have come to me about things that they have, but I just couldn't bring myself because people still laugh about wetting the bed. It's still a topic of “Ha‐ha! Do you wet the bed or something?”. – P18, female, age 17, night wetting and daytime urgency

Others feared that their problems would not remain confidential to their friendship group and that the whole school would find out about it.

I don't know [if I could tell my friends]. Because if it gets out, the whole school and they spread it, and then everyone will, like, picking on me and stuff about it. – P8, male, age 11, night wetting

A small number of participants had made the decision to take their friends into their confidence. Although they had been anxious about how to do this, or worried about the result, all of them reported positive experiences. In expanding their circle of disclosure, they experienced a sense of relief that their friends finally knew and that they no longer had to keep it a secret.

I was really relieved. It was a massive weight off my shoulders, because I'd been wanting to tell them for a while. And it was like, kind of, “How do I tell them? What do I say? What if they don't like me anymore? Oh my God.” And then I told them and they were like “All right then.” And I was like, “Oh, thank God for that,” you know, they weren't weirded out that much. – P15, female, age 14, daytime wetting

An older participant talked about why he had not told anyone at his school but had then made the decision to tell friends at his new college.

I was always afraid at school that people would make fun of you or they would just not want to be friends with you anymore. But I told this friend of mine at college and it was a complete like, I suppose it was like a complete non‐event, because he just, no reaction at all. He was just like “Yes, that's fine.” And I think it's more the fears inside my head, you know, and people's reactions. If I told lots of other people it would probably be exactly the same. – P12, male, age 19, night wetting

### 1b. Boundaries of disclosure: Teachers

Participants’ interactions with teachers were strongly influenced by whether or not they had disclosed their continence problem. As with their friends, deciding to disclose their continence problem to teachers, and which teachers, was a big decision. The power differential and nature of relationship with the teacher presented additional challenges. A few had approached their teacher(s) directly, although it was more common for participants to ask a parent or clinician to tell them by writing a letter to the school. Some participants were worried that their teachers would not take them seriously or believe it was a real problem. Bringing in a doctor's note helped to validate their problem and to make participants feel more comfortable talking to their teachers face to face.

Participants who did not want teachers to know about their problem said that they feared their teachers might not understand or even know that young people can experience continence problems. Young people were often concerned that teachers might treat them differently and this could cause unwanted attention from peers.

I'm just worried that if I did tell my teachers, then, they might, consequently, treat me a bit differently, like allowing me to go to the toilet but not other people. Then other people I'm not so close to [might find out]. – P3, female, age 18, daytime wetting

A few participants who had decided to tell their teacher(s) talked about the benefits of collaborating with the teacher to manage their continence problem. For example, sometimes the teachers allowed participants to access the toilet, and to do so with minimal fuss to avoid drawing attention to them.

Head of Studies emailed all the teachers to say “If he ever needs to go to the toilet, just let him […]” so whenever I needed to go to the toilet they let me go. Because I was embarrassed to say that I needed to go to the toilet, because if I had to go out two or three times in the lesson. – P19, male, age 13, soiling

### 2. Social consequences of avoidance

Participants’ interpersonal relationships and social life significantly suffered as a result of their continence problem. As most participants did not want anyone at school to know they had a continence problem, and even those who had told their friends did not want the rest of their peers to know, they spent considerable effort concealing it. Some felt like they were constantly deceiving people and living a lie.

It's the sheer fact that I have to lie constantly and I'm hiding. I feel like it's a huge secret because let's say, well explain, if I had to tell my friends “I wet the bed,” they'd be like “That's why you did this. That's why you had this excuse and that's why you didn't go there” and for people, everything would click into place. So I always feel a bit guilty lying. – P17, female, age 17, night wetting

To reduce detection or unwanted questions, participants reported that they often avoided certain social situations, or in more extreme cases, avoided making friends. If participants had an accident, this became even more challenging. One girl said she tried to keep a physical distance away from people when she had a soiling accident.

I try and stay a distance away so no one notices a smell or a stain or anything like that. – P13, female, age 11, soiling

Another boy talked about his frustrations of not being able to go on school trips.

There's a lot of things that I want to do, like staying overnight on school trips. And as much as that was sometimes possible with a lot of kind of careful planning, there were lots of times where, you know, I would just say “No I can't go,” but not really, and I always felt like I was having to think up excuses and think up reasons why I couldn't go to something. – P12, male, age 19, night wetting

As result of avoiding social situations to conceal their problem, some participants said that they felt isolated and alone.

It makes me feel really embarrassed and quite sad because if they're all playing a game together or something like that, and I have to go away from it because I don't want them to notice anything, I'll be a bit lonely. – P13, female, age 11, soiling

When I was younger how it made me feel is… I was isolated. Even if you're the most popular kid in the school, you do feel mentally quite isolated. – P11, male, age 14 daytime wetting

An older participant talked about the burden of carrying a secret and her worries about her current and future romantic relationships.

My boyfriend has no clue. My best friends have no idea that I have this underlying secret that affects my life in so many ways. They have no clue. So I think “if I get married I might [wet the bed]” and obviously married people share a bed. That's not news to anyone. So I'm like, “what am I going to do?” He might not want me there. He might kick me into the other room. – P17, female, age 17, night wetting

Other participants relayed upsetting messages of feeling abnormal or like an outsider.

…kind of feeling like an outsider, I suppose, feeling that you're not normal if that makes sense. – P12, male, age 19, night wetting

I wish it'd go away because it just feels like, when I am bad, I wish it would all go away and I wish that it would just stop and I could be like some of my friends and I might be normal. – P13, female, age 11, soiling

From these quotes, it is clear that young people attending school with continence problems are at risk of social isolation. Due to the stigmatization of incontinence, many reported feeling the need to hide their problem and frequently avoided social situations as a result. Not feeling able to disclose their problem to their friends also added to their sense of emotional isolation and feelings of being different.

### 3. Strict and oblivious gatekeepers

Many participants reported negative experiences relating to how teachers dealt with their continence problem. The most common adverse experience concerned restricted access to toilets during lessons. Even among participants whose teachers were aware of their issue, there were reports of encountering resistance when they asked to go to the toilet during class. This resulted in confrontations with the teacher, or in some cases, participants having a wetting/soiling accident during class.

Participant There was a time quite a while ago where there was this art teacher, she didn't let anyone go to the toilet in lessons. I went home and told me dad and he called the school and asked them to remind all the teachers.

Interviewer Yes, what happened with her?

Participant She wouldn't let me [go to the toilet] so I ended up soiling and it was really uncomfy. – P13, female, age 11, soiling

Continence problems are often viewed as a hidden disability as many young people who experience continence problems appear otherwise healthy and are adept at concealing their symptoms. A negative consequence of this is that some participants felt that their teachers often forgot that they had a problem and many were simply unaware of the wider issues these young people faced.

There's not a teacher that fully understands. They know how to deal with people with physical disabilities, like people that can't walk, but there is not a teacher that knows specifically about my problem and they don't really understand so much as I'd like them to. Some of the teachers just have to keep being told that if I'm asking something, or if I need to go to the toilet, they need to let me. Sometimes they forget and it's easy for them to forget, because I look normal, perfectly normal, so it's easy to not see that there's anything wrong with me, unless you actually know me. – P14, female, age 12, soiling

One participant had previously explained her continence problem to a teacher, only to be told subsequently by the same teacher that she should not have this problem at her age.

I told you I have got my [toilet access] card it is just a laminated thing. It was falling apart and when you need a new one you have to get it printed off and re‐laminated. I went to my pastoral manager who deals with everything like that and said I needed a new one. But she wouldn't give me one and said that it should be sorted by now and that we were going to have a meeting to discuss why I still needed it. – P10, female, age 16, night wetting and daytime urgency

### 4. Intimate actions in public spaces

The nature of continence problems meant that participants were forced to deal with intimate and personal issues within highly public spaces. The need to frequently use toilets and manage any wetting/soiling accidents meant that participants had to cope with private and intimate issues in a public manner.

All participants with daytime wetting, soiling, or urgency sometimes needed unrestricted toilet access throughout the school day; however, many schools restricted toilet access during lessons, presenting a significant challenge. In order to access the toilet, participants had either to agree this with their teacher by making them aware of their problem or, in some schools, apply for a medical access card (cards given to pupils for a variety of health reasons to permit them to leave lessons whenever required).

In my old school, my primary school, I had this card, and that's another way, I had a card. So when I needed to go to the toilet I would just take it out of my bag and just flash it at the teacher and then put it back away. So I could just walk out without having to ask any questions. – P19, male, age 13, soiling

The majority of participants reported positive experiences using their card; however, a few said they were worried about attracting unwanted attention and questions from their peers.

If someone went up to the teacher and asked to go to the toilet and the teacher says “No, because you are not allowed to leave,” then I did and I was allowed to leave because I have got my card people would be like “That is unfair, why is she allowed to go?” It made me feel awkward because I didn't know what to say. I used to say I had problems with my tummy. – P10, female, age 16, night wetting and daytime urgency

Almost all participants with daytime continence problems reported experiencing wetting or soiling accidents at school. The main issues they reported were changing pads and clothing, disposing of soiled pads or underwear, and cleaning themselves after an accident. School toilets offered minimal privacy or facilities to do this.

At the school that I'm at now, some of [the toilets] don't lock and some of them are alright now. They do lock but you just have to wait a bit. Once you're in there then it's like Quick get changed so no one knows that you've been in there a long time getting changed. – P4, female, age 14, daytime wetting

School toilets became places of fear and anxiety with the pressure to be as quick as possible and avoid discovery. Even making unusual noises were of concern.

The only think I do worry about is obviously because I put my wet ones in a nappy sack making a noise. It's just really weird because obviously if you hear a noise they'll think “What's going on?” then you come out I just don't want to be all embarrassed. – P16, female, age 15, daytime wetting

Girls reported that they often disposed of wet or soiled pads in sanitary bins, but that not all toilets had these facilities.

There's only a sanitary bin in one of the toilets, so I have to try and get there before anyone else does, otherwise I have to use the other one and then I'll be stuck. – P13, female, age 11, soiling

Boys do not have this option and either had to dispose of soiled items in the communal bin, risking discovery or confrontation, or keep them in their school bags.

Lots of participants described how they took ‘emergency packs’ to school including changes of clothes, wet wipes, and spare pads.

I keep knickers, if I'm wearing tights, I'll put tights in, I wear pads. Sometimes I pack a spare skirt, but not always, because sometimes I don't need it. It depends if I'm sitting down a lot or not, because if I'm sitting down, then I'll need one. If I'm not, then I won't. That's really all I pack. I usually take five pairs of knickers, because I don't know how I'll be that day. – P14, female, age 12, soiling

In my bag I carry around a little, small bag, which it has a spare pair on knickers and some nappy sacks. If I'm wet and need to change, then I can change. – P16, female, age 15, daytime wetting

One participant described her attempt to disguise a soiling accident. This particularly poignant account perhaps reflects how poor the toilet facilities were at her school, including the lack of privacy and challenges in washing and changing.

If it happens, I go into my bag and I get a pad out and I put it in my pants, but if it's too late and it's already in there, I'll get the tissue and put it on it and I'll put the pad on top so I can't feel anything but then there's the smell so I wash my hands with soap so hopefully it will overpower the smell of soiling. – P23, female, age 11, soiling

### 5. Interrupted learning

The need to use the toilet regularly meant that participants reported missing chunks of their lessons. Some of the participants felt they had missed so much that they were significantly falling behind and needing to catch up.

Rather quickly I can get left behind… I have to go out to go to the toilet, so it takes time that would have been time which other people would have been using to complete questions. – P6, male, age 15, daytime and night wetting

Some participants said that their teachers reacted negatively to them when they missed lessons.

I felt like I needed to go home, but I was scared because my science teacher, she can get a bit annoyed in the next lesson if you were away. Even if you caught up, if you don't understand something, she'll be like “Well, you should have been there then”. – P14, female, age 12, soiling

One participant said that she lacked the support to catch up and understand what she had missed.

[I want the teachers to understand] how it makes me feel if I miss lesson. They're like, “Oh, you should just get out of the lesson, it's fine, it's fine, everything will be sorted.” But, to me, it still doesn't feel sorted, because they're like “If it's your friends that are worrying you, we'll deal with them” I was like, “No. It's the work.” But then they're like “Oh you can catch up with that” but the teachers don't talk to you the same if you're like that, if you miss lessons. They won't talk to you the same, they assume, just because you've caught up that you understand it completely”. – P13, female, age 11, soiling

The need to visit the toilet during school also impacted on participants’ performance during tests and exams. Participants said they had lost time and were not able to complete questions, or, when toilet access was not permitted, their need to use the toilet reduced their concentration.

The other day, we mark ourselves and we get to see the mark scheme, and if I'd got that in a test, I would have only got a Level 3. I missed out on eight marks because I had to go to the toilet constantly. That's why I didn't get a high Level 4 or a secure Level 5 on my practice test. – P26, male, age 11, daytime and night wetting

I have to go to the toilet two or three times in between my lessons […] it's hard for me to get up and get out of the classroom because I don't want to miss anything… When I was doing my test I was really bursting but I had two or three questions to go and there was only five minutes left. When I went to the toilet, I got back and we had one minute left and I had three questions to do, so it kind of set me back on my marks. It's a good thing they were only practice. – P9, male, age 11, daytime and night wetting

Participants talked about feeling worried about upcoming tests or examinations, and feeling that their performance did not necessarily represent their ability or revision effort. For older participants, this had a significant impact on applying for college or university and added to their stress and anxiety levels.

I was finding doing the UCAT exams really hard, and I didn't know if I was going to be able to do it or not, which would have been a real shame because, obviously, I really want to study medicine. But it's essential for most medical schools that you do this exam. If I'd have got a lower result because I'd have had to have gone out in the middle of it to go to the toilet, and it's so time‐pressured, then, that could have cost me going to university. – P3, female, age 18, daytime wetting

## Discussion

The results of this study align closely with previous work conducted outside the United Kingdom on the experiences of school children with continence problems. These individuals all report less than satisfactory toilet conditions, conflicting rules regarding toilet access, and trepidation in disclosing their problem to teachers (Filce & LaVergne, 2015; Lundblad & Hellstrom, 2005; Lundblad *et al*., 2007). However, in addition to previous work, it highlights the social and academic challenges faced by this group. Furthermore, it provides a much needed evidence base to inform school policy within the United Kingdom.

Young people in this study reported experiencing anxiety and trepidation in disclosing their continence problems to their teachers and worried about their peers finding out. Although some had told their friends and had reported positive experiences, the vast majority had not. The underlying reason for this lack of disclosure was the perceived stigma of continence problems causing participants to worry about being bullied or teased, or being socially ostracized and seen as ‘different’. The stigma of continence problems has been previously documented in the literature within this age group (NICE, 2015a,2015b; Whale, 2016, Whale, Joinson, Cramer, & Wright, 2016). Young people with continence problems have been found to be at higher risk of peer conflict and teasing (Kelleher, 1997); in addition, students with chronic illnesses are frequently more socially isolated than their peers and are less preferred as playmates (Digirolamao, Quittner, Ackerman, & Stevens, 1997). Given these findings, it is not surprising that young people in the current study harbour worries about disclosing their condition.

Adolescence is one of the most sensitive stages of development in terms of identity and adjustment to physical changes (Lapsley, 2014). At this age, the desire to ‘fit in’ and be ‘normal’ is central to well‐being (Lovegrove & Rumsey, 2005). Whilst a large majority of adolescents will struggle with their identity or feel abnormal at one time or another, experiencing a continence problem creates a concrete point of difference and shame. Participants in this study used a variety of techniques in order to conceal their problem to avoid stigma and appear ‘normal’, much like Goffman's concept of ‘passing’ (1963). To appear ‘normal’, participants assume the identity of the majority, that is a young person without a continence problem, and are forced to maintain extreme vigilance in case their secret is revealed. Whilst this may prevent temporary upset and social discontent, it may also have a negative impact on identify formation and development. Individuals must live in a constant state of anxiety knowing their secret could be revealed at any time. In addition, by going to extreme lengths to conceal their real self, young people with continence problems are not able to fully accept themselves or their condition; a behaviour that Goffman suggests could lead to self‐contempt.

The stigmatizing nature of continence problems is also a hindrance in terms of accessing teacher support. Participants said that the majority of their teachers were not aware that continence problems could affect young people or that this is a real health problem. However, very few participants had told teachers about their problem or made them aware that they required additional support. This creates a dilemma in how best to provide appropriate support to young people with continence problems. Studies of other health problems affecting young people, such as epilepsy, cancer, and diabetes, have found that teachers are often ill informed about these conditions and how to deal with them (Bradbury & Smith, 1983; Charlton, Pearson, & Morris‐Jones, 1986; Checkryn, Deegan, & Reid, 1987; Court, 1994; Eiser, 1980; Eiser & Town, 1987; Lynch, Lewis, & Murphy, 1992). Teachers do, however, recognize this lack of knowledge and are keen to address it (Brook & Galili, 2001; Clay, Cortina, Harper, Cocco, & Drotar, 2004). There is a need to increase teachers’ awareness of the prevalence of continence problems in young people, and to provide guidance on how best to provide support at school. Indeed, previous work has highlighted that children with chronic illness feel that increased teacher understanding and support would benefit them in school (Mukherjee *et al*., 2000). However, if students are unwilling to tell their teachers about their problem, teachers will not be able to put this knowledge and support into practice.

Given the issues around disclosure and individual teacher support, attention may be better served in revising policies relating to toilet access at school and improving toilet facilities. Participants commonly reported how the school environment made managing their continence problems difficult, or exacerbated their problem. Successful management of continence problems often requires adherence to structured regimes of toileting and fluid intake, commonly combined with medication (Maternik *et al*., 2015; NICE, 2010; von Gontard, 2012a, 2012b). To do this, young people need unrestricted access to toilets during the school day. However, for participants in this study, toilet access was predominantly restricted to break times, with requests to use the toilet during lessons frequently met with resistance from teachers. There was also a lack of facilities for young people to clean themselves after a wetting or soiling accident and for discreetly disposing wet or soiled pads. School toilets often had no locks on cubicle doors and only provided sinks and bins in the communal areas. This made using the toilet a potentially risky activity for participants, as they felt vulnerable and highly anxious about being discovered. Some participants avoided using the toilet altogether, even if they had an accident.

Under the Children and Families Bill (2013), schools have legal requirement to provide appropriate support for children with health problems; however, from these results, it is clear that the needs of children with continence problems are not being met. These findings are supported by a review of UK school toilets conducted in 2013 that reported restricted toilet access, lack of door locks, smelly and unhygienic toilets, fear of bullying, lack of sanitary bins, and restricted time to use the toilet (Burton, 2013). Other smaller scale international studies show high levels of toilet avoidance due to substandard facilities (Lundblad & Hellstrom, 2005; Vernon *et al*., 2003; Filce & Bishop, 2014). Several campaigns have been launched to tackle this problem including ‘Bog Standard’ (2004), and the more recent ‘Right to Go’ spearheaded by the paediatric continence charity ERIC (2014). These campaigns provide guidance about appropriate policies and procedures in place to support young people with continence problems in educational settings and on legislation relating to school toilet best practice guidelines. The campaigns also give advice on how to support students with continence problems, such as providing access to single lockable toilet cubicles with a private sink and changing space. The existence of campaigns such as these highlights the significance of this problem, and the work that needs to be done to improve the school experience of young people with continence problems.

A concerning finding from this study is the impact of continence problems on learning and academic performance and the apparent disadvantages faced by young people with continence problems. Participants commonly reported that their lessons were disrupted due to frequent toilet visits and those with severe daytime continence problems reported leaving the classroom 3 or 4 times during a lesson to use the toilet. This finding is consistent with other studies on school experiences in young people with continence issues and other chronic health conditions which report higher rates of absenteeism in these populations (Filce & LaVergne, 2011, 2015; Sato *et al*., 2007; Wodrich & Cunningham, 2008). Absenteeism, even for part of a lesson, creates reduced opportunities for learning, asking questions, and teacher contact. Students who are frequently absent have a high risk of falling behind and poor academic performance (Carroll, 2010; Needham *et al*., 2004). Participants in this study said they often fell behind during lessons, or missed out on time during examinations due to needing to use the toilet. When toilet access was not allowed, their concentration was negatively affected either by the need to go to the toilet, or by feeling anxious about a possible accident. This is a particularly troubling finding as academic performance in secondary school had a direct impact on further education opportunities, and future employment (Boliver, 2013; Hemsley‐Brown, 2014; Kuncel, Hezlett, & Ones, 2004). In the United Kingdom, there are policies in place for students with health problems to be considered for additional support or special arrangements during examinations (Equality Act 2010). This could involve extra time during examinations or supervised toilet breaks. It is important that young people with continence problems are aware of these policies so that they are not disadvantaged by their health problem.

### Strengths and limitations

This study provides evidence of the only detailed qualitative study carried out with young people with continence problems in the United Kingdom and extends the age range previously studied outside the United Kingdom. It includes young people with a range of continence problems including daytime wetting, night wetting, soiling, or a combination of these. This work adds to a much needed evidence base on the impact of paediatric continence problems and provides the first set of qualitative data on UK school experiences. In‐depth interviews provide rich data and a diverse range of experiences on which to build future work and inform school policy. The wider research team includes paediatric continence specialists with years of clinical experience of listening to this group. Whilst the stigma of incontinence has been previously documented, the extent and level of stigma described by the participants is powerful and surprising.

With regard to limitations, only a small number of participants with soiling problems were included in this study because of challenges in recruiting these young people. Due to differences in toileting needs of young people with bowel problems, it is likely they may have different experiences to those with bladder problems. All participants were attending mainstream secondary school or, in one case, college. It is possible that some young people with continence problems attend more specialized schools for children with physical conditions or additional needs. It would be beneficial to compare the experiences of these groups to see whether further lessons could be learned about supporting these pupils. This study could not explore cultural differences in young people's experiences of continence problems as all but one of the participants identified as White British. Socioeconomic background could also impact on experiences of young people with continence problems, but these data were not collected in the current study.

### Conclusion

Disclosure of continence problems at school to both friends and teachers was rare, due to the perceived stigma and fears of bullying and social isolation. The lack of disclosure to teaching and education staff creates challenges in how best to support these young people. Due to the needs of young people with continence problems to have full access to private toilet facilities during the day, schools should have inclusive toilet access policies and improve toilet standards. Addressing the challenges faced by young people with continence problems at school could help to remove the barriers to successful self‐management of their symptoms. It is particularly concerning that some young people in this study felt that they are at higher risk of academic underachievement. Increased support at school is needed to enable young people with continence problems to achieve their academic potential.

## Conflict of interest

All authors declare no conflict of interests.

## Appendix 1 ROCCA study topic guide

**Table:**

Section	Activity	Warm‐up questions	Continence questions	Notes
About you ice‐breaker	() ()	The first activity is to get to know a bit more about you and what you like doing. You can use the page to draw or write or just talk if you like. What do you like to do in your spare time? Do you have any hobbies? What do you do at weekends? What do you do in the evenings? After school/work?		
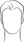
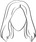
Food and drink	()	What food and drinks do you like? Are there any foods you really don't like? How about meal times in your house? Do you have a set routine or does it change a lot? How about at school? Can you take drinks into class?	Are there any foods or drinks you avoid to help with your continence? Do you drink during the day? Have you tried eating and drinking different things?	
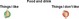
Who is in my life	()	Who do you spend most of your time with? Why do you like spending time with them? Do you have a best friend? Do you chat to people online? Chat rooms, Facebook, twitter	Do you have anyone in particular you talk to about your problems? Have you told any of these people about your continence problems? What words or phrases do you use? What have you told them? / Why not? Do any of these people help you with your problem? What do they do? Boyfriend/girlfriend? Would you do different activities if you didn't have a continence problem?	
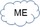
Who lives in my house	()	So we've talked a bit about your friends and who you spend time with, how about your family? Who do you live with? Do you share a room with anyone? Where do you spend most of your time? Do you have friends over after school or at the weekend?	Do you stay over at friends’ houses or have friends to stay over? Does your continence affect this? Do you worry about it? Do you take any precautions? Plans?	
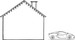
A typical day	()	Tell me about a typical day during the week… Tell me about what you do in the morning What time do you normally wake up? *School college* What time do you go to school/college? What time do you normally get home? How about the weekends? Now you're at college / you've left school, has this made a difference to your continence problem? *Work* What time do you go to work? Do you enjoy your job? Can you use the toilet whenever you need to? *Other*	*If wet/soil at night…* How often do you have a wet night? What happens when you wake up? What do you do to manage it? Do you change your own sheets? Are there times when the problem is worse? How do you feel after a wet night? *If wet/soil during day…* What happens if you have an accident during the day? Do you take spare clothes or underwear? Is there somewhere you can change? Do you worry that people will notice? Are there times of the day it is worse? Are there certain situations that make it worse?	
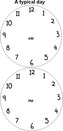
School / college (if relevant)	()	Tell me a bit about your school/college What lessons/subjects do you enjoy? Are there any lessons you don't like? What are the teachers like? Are there any teachers you like or you can talk to? What are the school toilets like? Can you go to the loo when you need to? Does you school have access cards? Do you play any sport or have PE lessons? Can you use the changing rooms or showers if you need to? What do you study at college? Do you go every day?	What happens if you have an accident at school? Can you get changed if you need to? Do your teachers know about your problem? How do they know? Did you mum/dad talk to them? Can you talk them about it? Do you want to? Do any teachers help you if you have a problem/accident?	
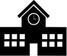
Practical issues		Who does the washing / laundry in your house? Where is the washing basket kept? Can you do your own washing? Do you share a room with anyone?	Do you have any strategies for managing your problem? What do you do if you have accident outside the house? What do you parents do when you have an accident? What do you do with your clothes / bedding when you have an accident? Do you take spare clothes with you? Do you wear pads? When do you have the most problems? Are there times when it is better? Do people notice when you have a problem? What do they do?	
My life	()	What important/ memorable things have happened in your life?	When did you first realise you had a continence problem? Did it get better/worse at any time?	
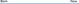
Thoughts and feelings	()		How does your continence problem make you feel? What do you do when you feel xxxx? What do you think about your continence problem?	
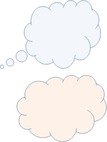
Information and support	()		Do you have any support for your continence problem? What kind of advice have you had about your problem? When did you start looking for advice or support? Was it you or your parents that started looking? Do you know about ERIC? How did you find out about ERIC? Do you know anyone else with the same problem? What do you think about doctor appointments? Do you find them helpful? Do you find the doctors easy to talk to? Do you worry about appointments? What advice would you give someone with the same problem? Are there any tips or tricks that work for you? Is there anything you have tried that doesn't work? Is there anything else you would say to them?	
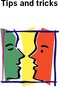
Goals and aspirations	()	If you imagine yourself in 5 years’ time, what would you like to be doing? Who are you with? How are you feeling? How about in 10 years’ time? Do you want to have a job? What would it be? Where would you like to live? Would you like to take a gap year? Would you like to go travelling? Where would you like to go?		
